# Variance of radiographical alveolar bone mineral density by the anatomical morphology of mandibular bone

**DOI:** 10.1016/j.heliyon.2022.e11507

**Published:** 2022-11-12

**Authors:** Akitoshi Katsumata, Kiyomi Kohinata, Yuka Esaki, Michio Kawai

**Affiliations:** a Department of Oral Radiology, Asahi University School of Dentistry, Japan; b Asahi University Medical and Dental Center, Japan; c Japan Oral Clinic, Japan

**Keywords:** Intraoral radiograph, Alveolar bone, Bone mineral density (BMD), Mandible

## Abstract

**Purpose:**

Evaluating the bone mineral density (BMD) of alveolar bone is useful for dental treatments. The DentalSCOPE is an image analysis system developed to evaluate the BMD of alveolar bone. The aim of this study was to evaluate the relationship between cross-sectional anatomical size and BMD value.

**Materials and methods:**

Thirty-four subjects (adult dental patients and volunteers) participated in this study. Intraoral radiographs of the mandibular molar region were acquired. Using DentalSCOPE software, three to four line-shaped regions of interest (ROIs) were obtained in the alveolar septum region. Cross-sectional CT images of mandible at the same position to above mentioned line-shaped ROI was reconstructed from subject's dental CBCT images. The measurements were performed using cross-sectional CT images and compared with BMD value.

**Results and discussion:**

For stepwise multiple linear regression analysis, the buccal-lingual width of the mandibular body (mandible width) and the CT value of the cancellous bone were adopted as explanatory variables that affected the BMD of the mandible. The BMD value increased by 20 mg/mm^2^ when the mandible width increased by 1 mm, and the BMD value increased by 5 mg/mm^2^ when the CT value of the cancellous bone increased by 1%.

**Conclusion:**

In the clinical application of alveolar bone BMD, the effect of the anatomical morphology of alveolar bone should be taken into consideration.

## 1 Introduction

Bone mineral density (BMD) examination is important for the diagnosis of osteoporosis. Primary osteoporosis is diagnosed based on BMD level in the lumbar spine and femur bone [1]. The dual-energy X-ray absorptiometry (DXA) system is used in the lumbar spine and femur bone; however, it is difficult to apply the DXA system to the jaw bones. In the field of dentistry, conventional microdensitometry techniques have been used to measure BMD [2, 3, 4, 5], which have been proposed to identify osteoporosis patients and to evaluate alveolar bone condition for periodontic, endodontic, and surgical treatment [3, 4, 5]. Sakagami et al. measured the X-ray film density of alveolar bone region to evaluate the change after scaling/root planning treatment [3]. Takaishi et al. evaluated the change of the alveolar bone BMD in the patients with anti-resorptive agents-related osteonecrosis of the jaw (ARONJ) and evaluated the osteo-sclerotic change of lesion [4].

In the era of film-based X-ray imaging, it was too complicated to measure the optical density of a film image and calculate BMD value. Therefore, BMD measurement was not applied as a routine examination in dental practice. Recently, the photo-stimulatory phosphorescent substance (PSP) digital intraoral imaging system has become popular, and we have been able to develop an easy-to-use BMD measurement system for the alveolar bone [6].

This new system improved the previous microdensitometry techniques which applied for intraoral radiographs to fit the digital images. The improvements include a calcium carbonate reference objects with a defined BMD value and computer program that enable to obtain the BMD value of an arbitrary area set on the image.

Regarding the long bones of arms and legs, it has been reported that the thickness of cortical bone affects BMD value [7, 8, 9, 10]. However, whether the buccal and lingual cortical bone thickness affects the alveolar bone BMD or not has not been studied. In the present study, we measured cross-sectional anatomical size and CT value in the mandibular molar region on cross-sectional CBCT images and compared with BMD values.

## 2 Materials and methods

This study was performed with the approval of the Ethics Committee in Asahi University School of Dentistry (approval no. 32028). This study was conducted according to the principles expressed in the Declaration of Helsinki. Ten adult dental patients and 24 adult volunteers participated in the study (males n = 16, females n = 18; average age 42.5). Informed consent was obtained from all participants in the study.

### 2.1 BMD measurement

**Figure 1:**
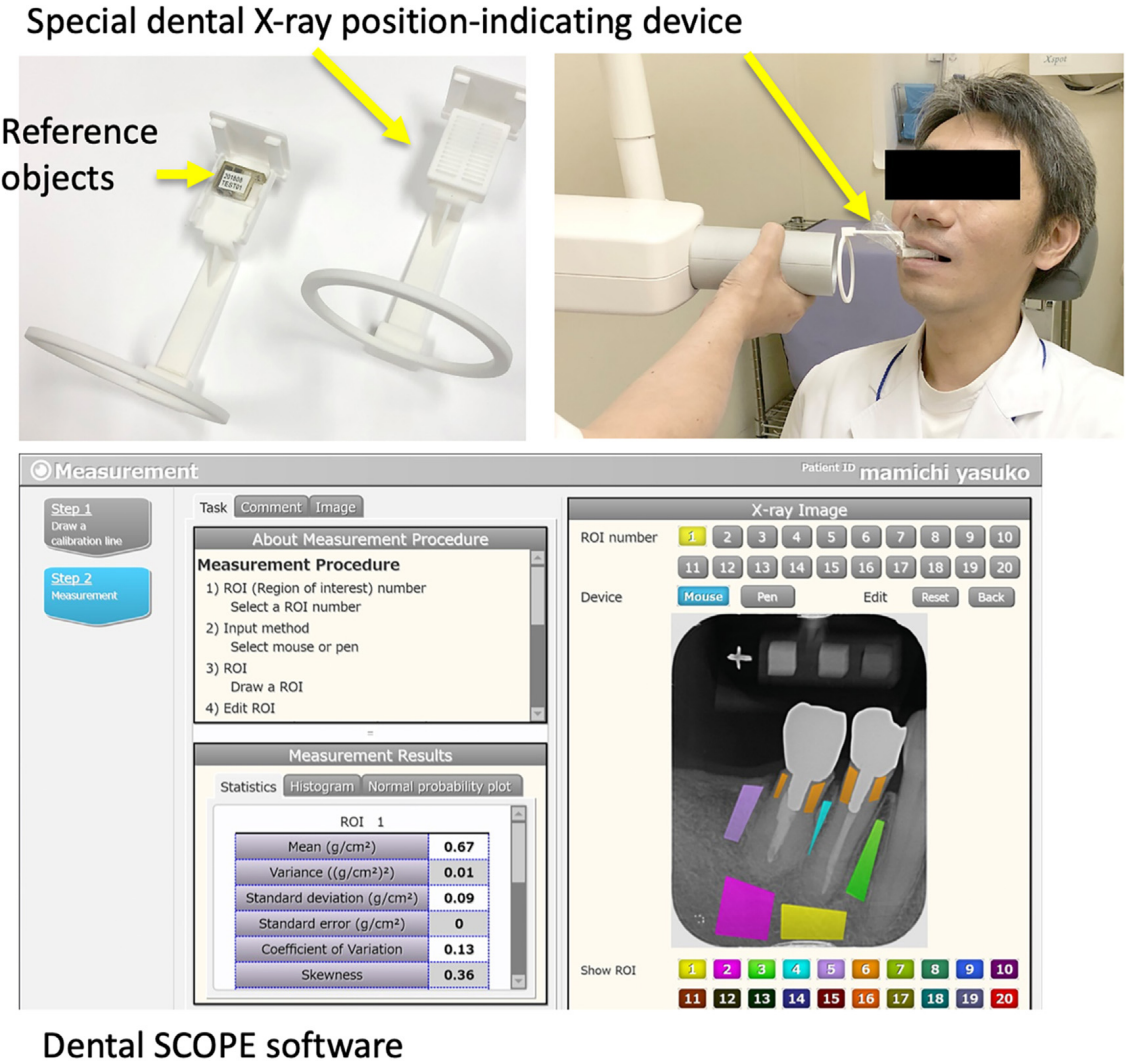
Dental SCOPE system. The BMD measurement system consists of a reference object, an X-ray indicating device, and software.

The DentalSCOPE (Media Corporation, Tokyo, Japan) system was used to evaluate the BMD of alveolar bone (Figure 1).

BMD measurement is based on conventional microdensitometry techniques. The DentalSCOPE consists of a special intraoral X-ray position-indicating device and software. This indicating device was designed to embed reference objects. The reference objects were made of 20, 60, or 100% calcium carbonate. DentalSCOPE software measures the image density of the reference object automatically and calculates the BMD value of the arbitrary region of interest (ROI).

**Figure 2:**
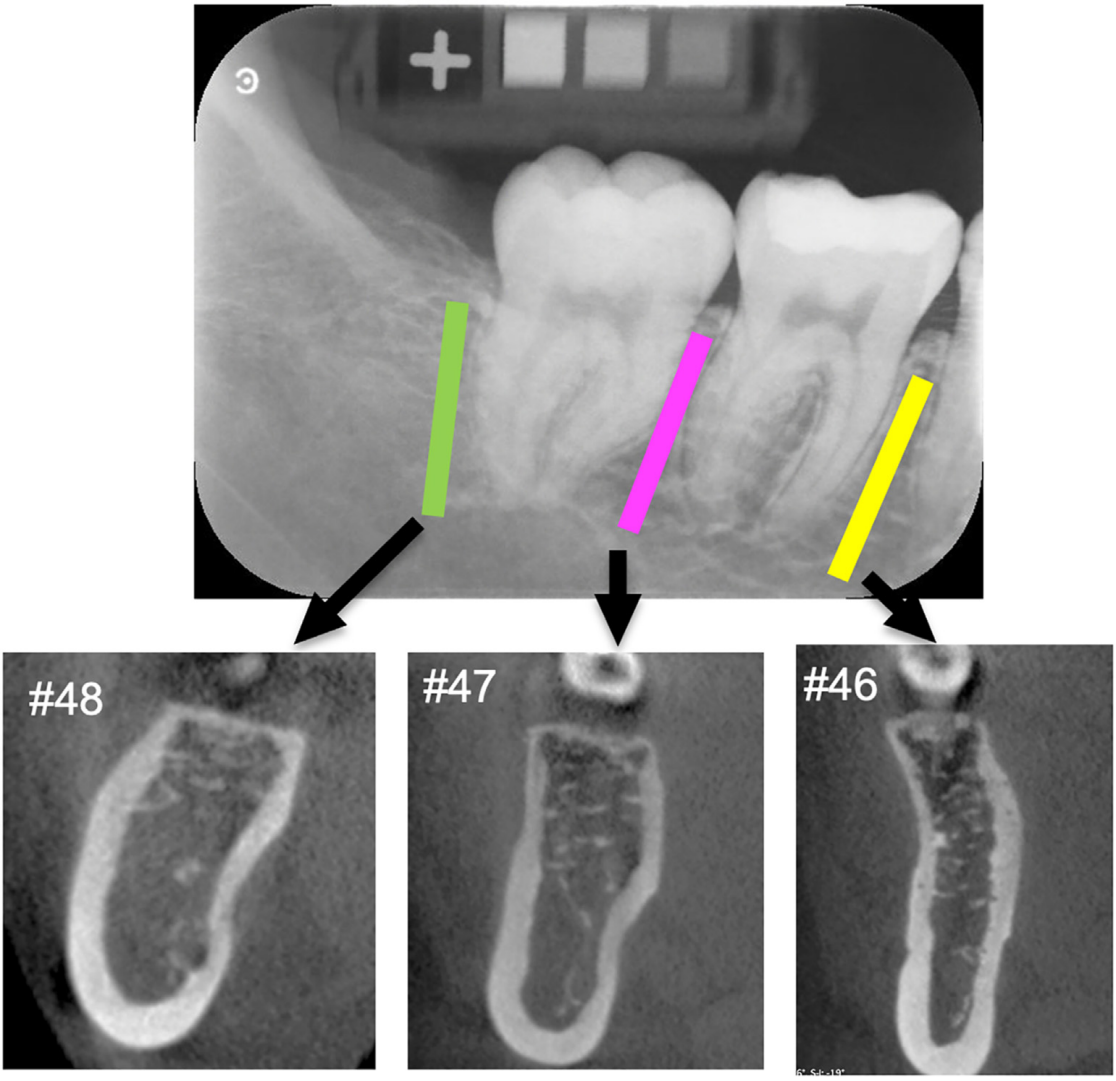
ROIs in an intraoral X-ray image and multi-planer reconstruction CBCT image of the same region. Three ROIs were set in an intraoral X-ray image to measure BMD. Cross sectional CBCT images were reconstructed in the tooth number 46, 17, and 48 regions.

Intraoral radiographs of the mandibular molar region were acquired using X-ray irradiation at 70 kV and 6 mA. The time of irradiation was set as 0.2 s. Digital X-ray images were obtained using the Carestream CS 7600 (Carestream Co., Rochester, NY) PSP imaging system. X-ray image data was input into DentalSCOPE software with JPG or BMP file format. The BMD value for the line-shaped ROI was drawn on the alveolar septum region. Three or four ROIs were placed on the interdental alveolar bone of one X-ray image (Figure 2A).

### 2.2 Cone-beam computed tomography (CBCT) imaging and measurement

**Figure 3:**
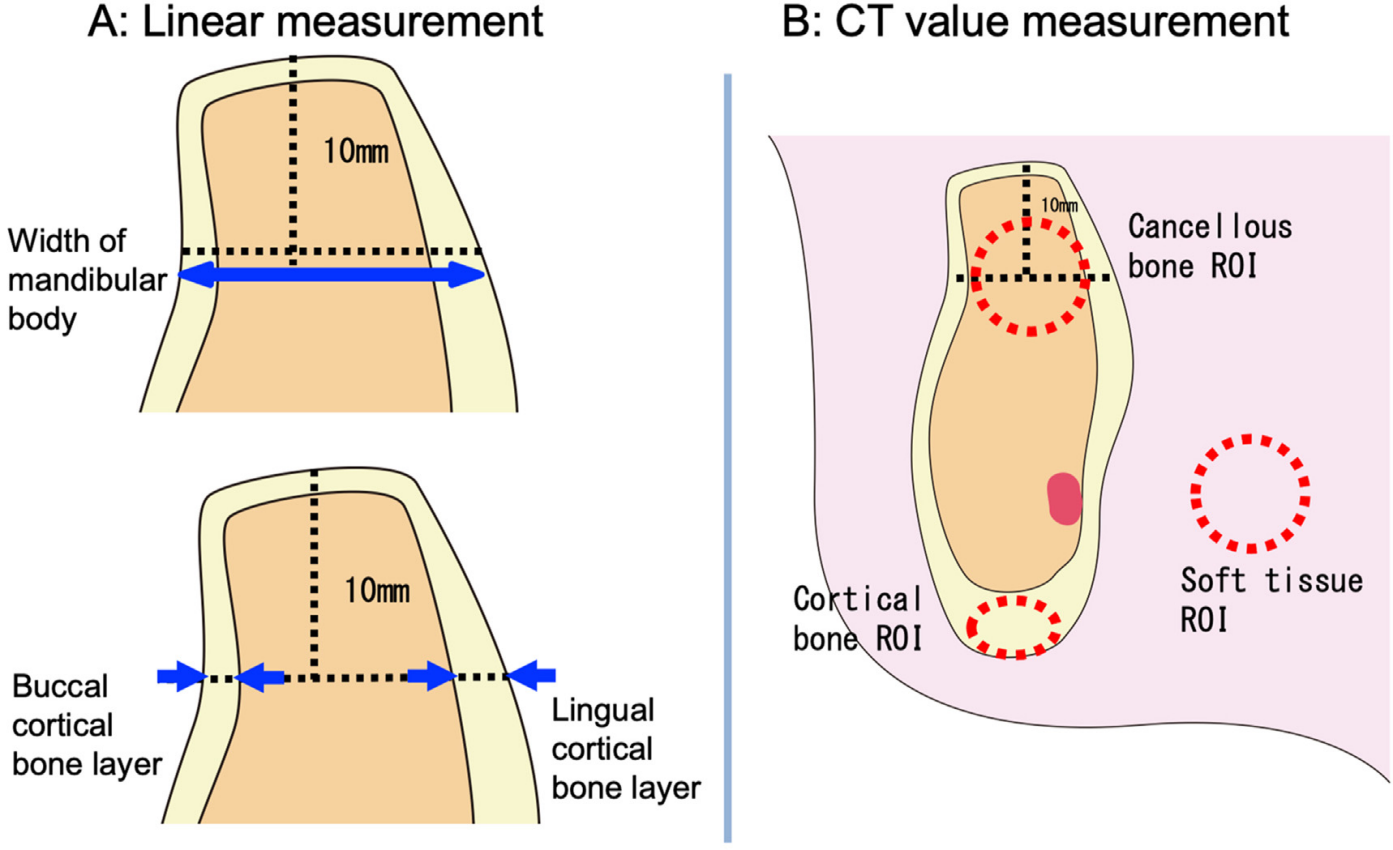
Linear and CT value measurement items. The buccolingual width of mandible and the thickness of buccal and lingual cortical bone layer was measured (A). The CT value of cancellous bone, cortical bone, and soft tissue regions were measured (B). The relative CT value of the cancellous bone was calculated when the CT value of the cortical bone is 100 and the CT value of any tissue is 0.

The Alphard Vega 3020 (Asahi Roentgen Co., Kyoto, Japan) and QRmaster-H (Takara Belmont Co., Osaka, Japan) systems were used for CBCT image acquisition. The height and diameter of the field of view were 51 mm and 51 mm, respectively. The size of a voxel was 0.1 × 0.1 × 0.1 mm. Image analysis software (Osirix MD; Pixmeo, Geneva, Switzerland) and Multi-planar reconstruction (MPR) function was used to reconstruct cross-sectional CT images of the mandible bone and dental arch. The cross-sectional position was set as the same position to attain the BMD of the line-shaped ROI (Figure 2B). The thickness of MPR slice image was set as 1.0 mm. The following linear measurements and CT value measurements were performed in cross-sectional CT images (Figure 3A, B):

#### 2.2.1 Width of mandibular body

• Thickness of buccal and lingual cortical bone layer
• Total thickness of both buccal and lingual cortical bone
• The percentage of the cortical bone within the width of the mandibular body
• CT value of the cancellous bone region

Regarding the CT value of the cancellous bone region, as the dental CBCT machines do not employ the Hounsfield Unit as the standard CT value, a special relative value was used. The coefficients for determining the special relative values were obtained by drawing circular ROIs in the cancellous bone, cortical bone, and soft tissue regions and measuring the CT values (Figure 3B). The relative CT value of the cancellous bone was calculated with the CT value of the cortical bone as 100 and the CT value of any soft tissue as 0.

### 2.3 Statistical analysis

The logistic regression analysis was performed using a statistical system (SPSS 23, IBM Japan, Tokyo) to study which of the morphological measurement item of the mandible affected the BMD value. The BMD value is the objective variable, and the above-mentioned CT measurement items are the explanatory variables. A stepwise variable selection procedure was used. The null hypothesis that the multiple correlation coefficient of each explanatory variable for the obtained multiple regression equation was 0 was verified. A variable with a p value of 0.5 or less determined from the correlation coefficient was considered to have explanatory power. A scatter plots with BMD values on the x-axis and CT measurement values on the y-axis were created.

## 3 Results

**Figure 4:**
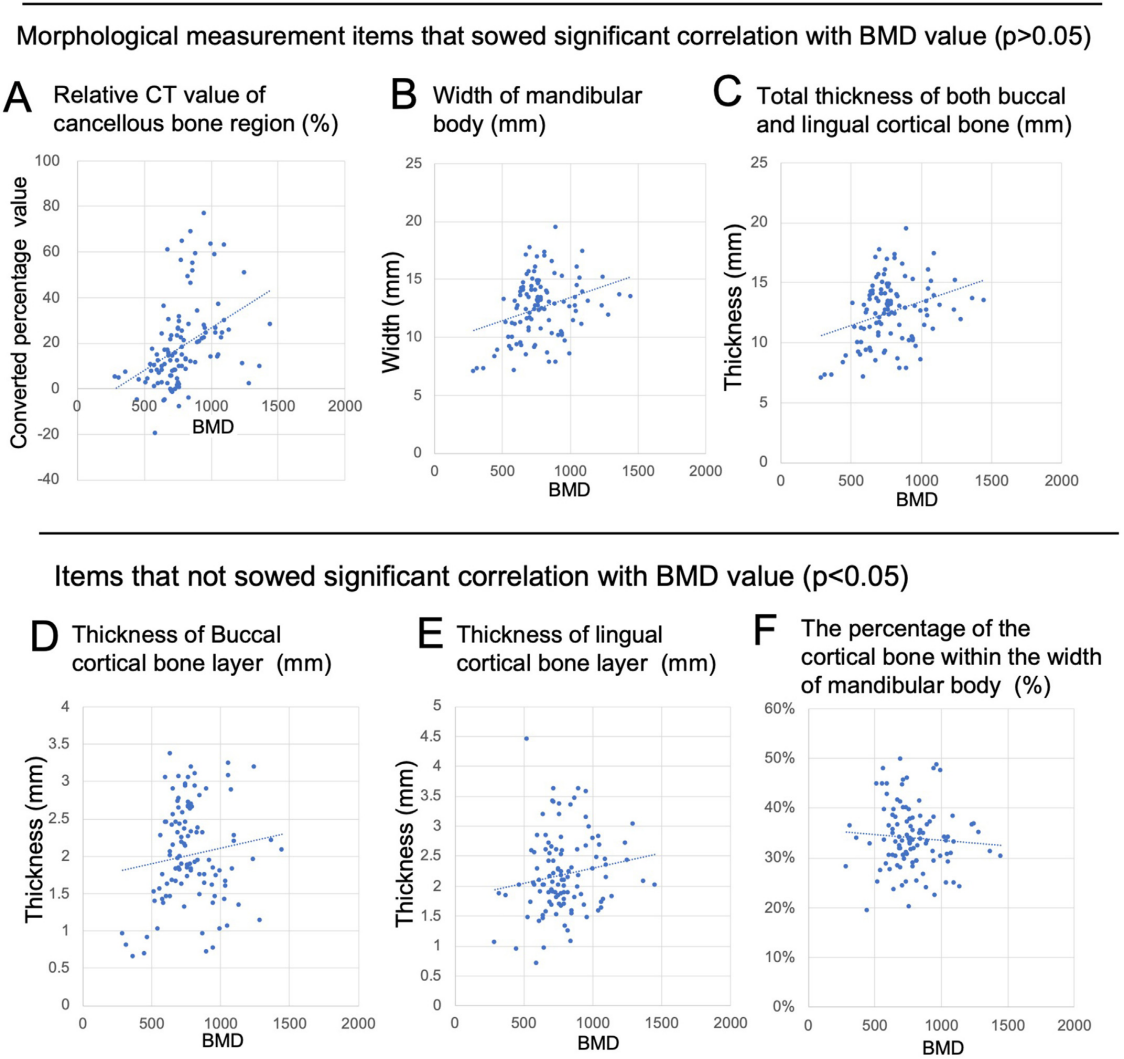
Scatter plot relationship between BMD value and CT measurements. A, B, and C showed a significant correlation with the alveolar bone BMD. D, E, and F Did Not Show a Significant Correlation with the Alveolar Bone BMD.

BMD values of line-shaped ROIs were measured in a total of 108 alveolar bone regions. Linear and CT value measurements were performed in the same number of cross-sectional CT images of the mandible. Figure 4 shows the scatter plot relationship between BMD value and CT measurements. Table 1 shows the Pearson's correlation coefficient between BMD value and CT measurement items. As a result of stepwise multiple linear regression analysis, the correlation coefficient of relative CT value of the cancellous bone region (0.504) was significant at 1%. The buccal-lingual width of the mandibular body (mandible width) and the total thickness of both buccal and lingual cortical bones were significant at 5%. The correlation coefficient was 0.292 and 0.186 respectively.

**Table 1:** Pearson's correlation coefficient between BMD value and CT measurement items.

Morphological measurement item	Pearson's correlation coefficient
Width of mandibular body	0.292∗
Thickness of buccal cortical bone layer	0.147
Thickness of lingual cortical bone layer	0.113
Total thickness of both buccal and lingual cortical bones	0.186∗
The percentage of the cortical bone within the width of the mandibular body	−0.066
CT value of the cancellous bone region	0.504∗∗

∗ Significant at 5% (based on calculated p value).

∗∗ Significant at 1% (based on calculated p value).

A multiple regression equation was created to calculate the BMI value. The partial regression coefficients of the relative CT value of the cancellous bone region and the mandible width, which showed a large Pearson's correlation coefficient, were 20.73 and 4.64, respectively. This equation revealed that the BMD value increased by 20 mg/mm^2^ when the mandible width increased by 1 mm, and the BMD value increased by 5 mg/mm^2^ when the CT value of the cancellous bone increased by 1%.

## 4 Discussion

Digitization of dental X-ray images has facilitated quantitative diagnostic imaging, which was difficult in the era of film-based X-ray imaging [11]. Thus, it has become possible to easily measure the distance, area, or image density using a computer. The Dental SCOPE system for measuring alveolar bone BMD from image density of intraoral radiographs used in this study is to say a gift of technological advancement of the digital age. Our proposed BMD measurement method has several advantages. The exposure dose for an intraoral radiography is estimated as approximately 1.0 mGy (air kerma) [12]. This irradiation is much lower than other modalities such as dental CBCT and DXA.

Although DXA is the most reliable and commonly used radiological modality to measure BMD [13, 14], it was difficult to develop a special DXA system to measure the BMD of alveolar bone. To measure the BMD of bone that is surrounded by thickened soft tissue, such as the femur, accurately, DXA is required to reduce the influence of soft tissue. The thickness of the soft tissue surrounds the femur is large and has varies greatly from person to person. On the other hand, the soft tissue surrounding the alveolar bone is thin and the individual difference is small. In the intraoral imaging, an X-ray beam does not need to pass through thickened soft tissue to reach a PSP imaging plate that is placed in the lingual or palatal side of a patient's dental arch. Therefore, intraoral imaging has less necessity to apply DXA than lumbar spine and femur bone. These anatomical features of the alveolar region and technical feature of the intraoral X-ray imaging can be considered suitable for applying conventional microdensitometry technique to measure alveolar bone BMD.

Positive relationship between the BMD value and the thickness of cortical bone has been reported in the tibia, femur, and upper limbs [7, 8, 9, 10]. As the mandibular belongs to the long tubular bone, it is natural that the thickness of cortical bone is correlated with the BMD value. Taguchi et al. studied the relationship between the width of the inferior cortex of the mandible on panoramic radiographs and the BMD of the systemic bone [15, 16]. And they established that the width of the inferior cortex of the mandible is a useful criterion to screen possible osteoporosis patients.

On the other hand, this study revealed that the correlation coefficient between the BMD of the alveolar bone and the thickness of buccal and lingual cortical bone was lower than 0.2. This may be due to anatomical characteristics of the alveolar bone region as it has a porous and thin cortex and cancellous bone with many trabeculae. The cortical bone layer of the long bones of the limbs has a uniform thickness all around, but the mandible is different. In the jaw bones, the cortical bone in the alveolar region is very thin compared to the inferior cortex of the mandible. The result that cortical bone thickness in the alveolar region does not affect BMD is an understandable explanation for the significant correlation coefficient (0.504) between CT value of the cancellous bone and BMD. Aleksova et al. reported that the trabecular bone score (TBS) which obtained by textural analysis and representing the amount of trabecular bone correlated with BMD [17]. The amount of bone trabeculae may affect the BMD value of the alveolar bone. The sclerotic change of alveolar cancellous bone often occurs due to periodontal and periapical disease. In the clinical use of BMD measurement, the effect of periodontal inflammatory disease should be taken into consideration on the measurement results.

We used a special relative value instead of the Hounsfield Unit for CT value measurement of the cancellous bone region. Minimal X-ray irradiation is an advantage of limited area CBCT imaging; however, the CT value obtained revealed high variability [18]. Thus, in the present study, we used a special relative value. If the same measurements were performed on a whole-body CT image with the Hounsfield Unit available, a better correlation than the present study may be yielded between the CT value of the cancellous bone and the BMD values of the alveolar bone.

In the present study, the width of the mandible body revealed a distinct correlation between the BMD values of the alveolar bone; therefore, when comparing BMD values between patients, the region to be measured should be unified. It has been known that highly reproducibility intraoral imaging is difficult. Although the DentalSCOPE system equips a special intraoral X-ray position-indicating device, careful setting of X-ray exposure is necessary for operator to obtain accurate and reproducible intraoral image. Another complication point of the DentalSCOPE system is the need for image density calibration procedure for the adjustment of each PSP intraoral imaging system used. Improving these disadvantages are the important issue for the future.

Further studies are required to establish the distinct clinical usefulness of BMD of the alveolar bone. Patients who receive bone resorption inhibitors, such as bisphosphonates and denosumab, have a risk of developing anti-resorptive agents-related osteonecrosis of the jaw (ARONJ) [19]. Because osteoporosis medicines affect the alveolar bone, the increase of alveolar bone BMD in patients with ARONJ has been reported [4]. It may be possible to predict ARONJ by means of continuous observation of alveolar bone BMD. Zamani et al. reported that not only osteoporosis medicine but also the lithium carbonate medicine used for psychiatric patients may increase the BMD [20]. It may be an interesting topic to investigate the effect of lithium salt administration on BMD of alveolar bone.

## 5 Conclusion

The relationship between the effect of the cross-sectional anatomy in the CBCT image and the BMD value of mandibular alveolar bone was evaluated. The alveolar bone BMD showed significant correlation coefficient with mandible width (0.292) and CT value of the cancellous bone (0.504). The correlation coefficient between the alveolar bone BMD and the thickness of buccal and lingual cortical bone layer was lower than 0.2.

## Declarations

### Author contribution statement

Akitoshi Katsumata: Conceived and designed the experiments; Analyzed and interpreted the data; Contributed reagents, materials, analysis tools or data; Wrote the paper.

Kiyomi Kohinata; Yuka Esaki: Performed the experiments.

Michio Kawai: Analyzed and interpreted the data; Contributed reagents, materials, analysis tools or data.

### Funding statement

This research did not receive any specific grant from funding agencies in the public, commercial, or not-for-profit sectors.

### Data availability statement

Data will be made available on request.

### Declaration of interest’s statement

The authors declare no conflict of interest.

### Additional information

No additional information is available for this paper.

## Appendix A Supplementary data

R3 Figures R3 Figures
