# Supplementary material for: Variance of radiographical alveolar bone mineral density by the anatomical morphology of mandibular bone
Source: Heliyon. 2022 Nov 12;8(11):e11507. doi: 10.1016/j.heliyon.2022.e11507 (PMC9676521; doi:10.1016/j.heliyon.2022.e11507)
Supplement: R3 Figures [file mmc1.pptx]

## Slide 1
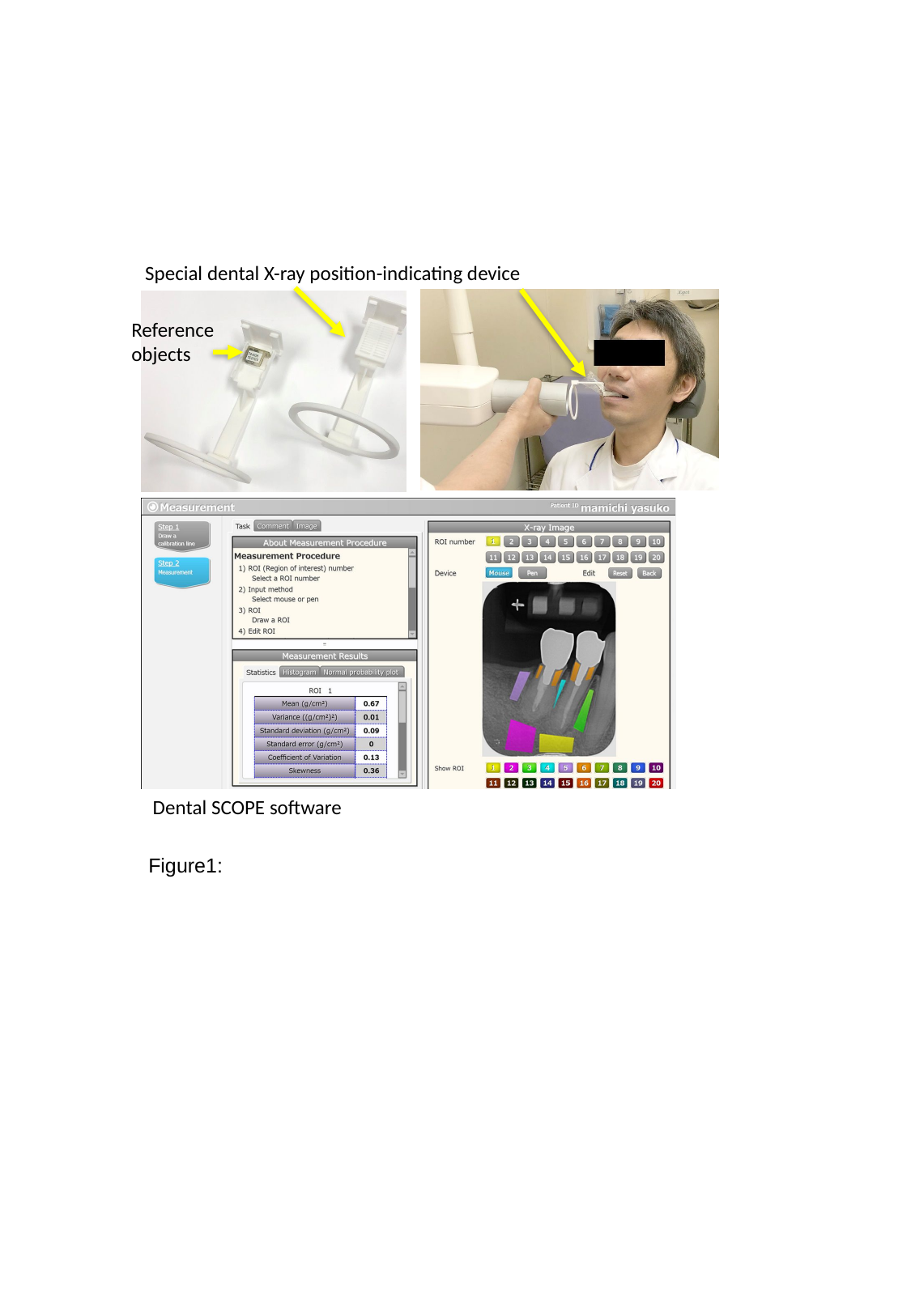

Special dental X-ray position-indicating device
Reference objects
Dental SCOPE software
Figure1:

## Slide 2
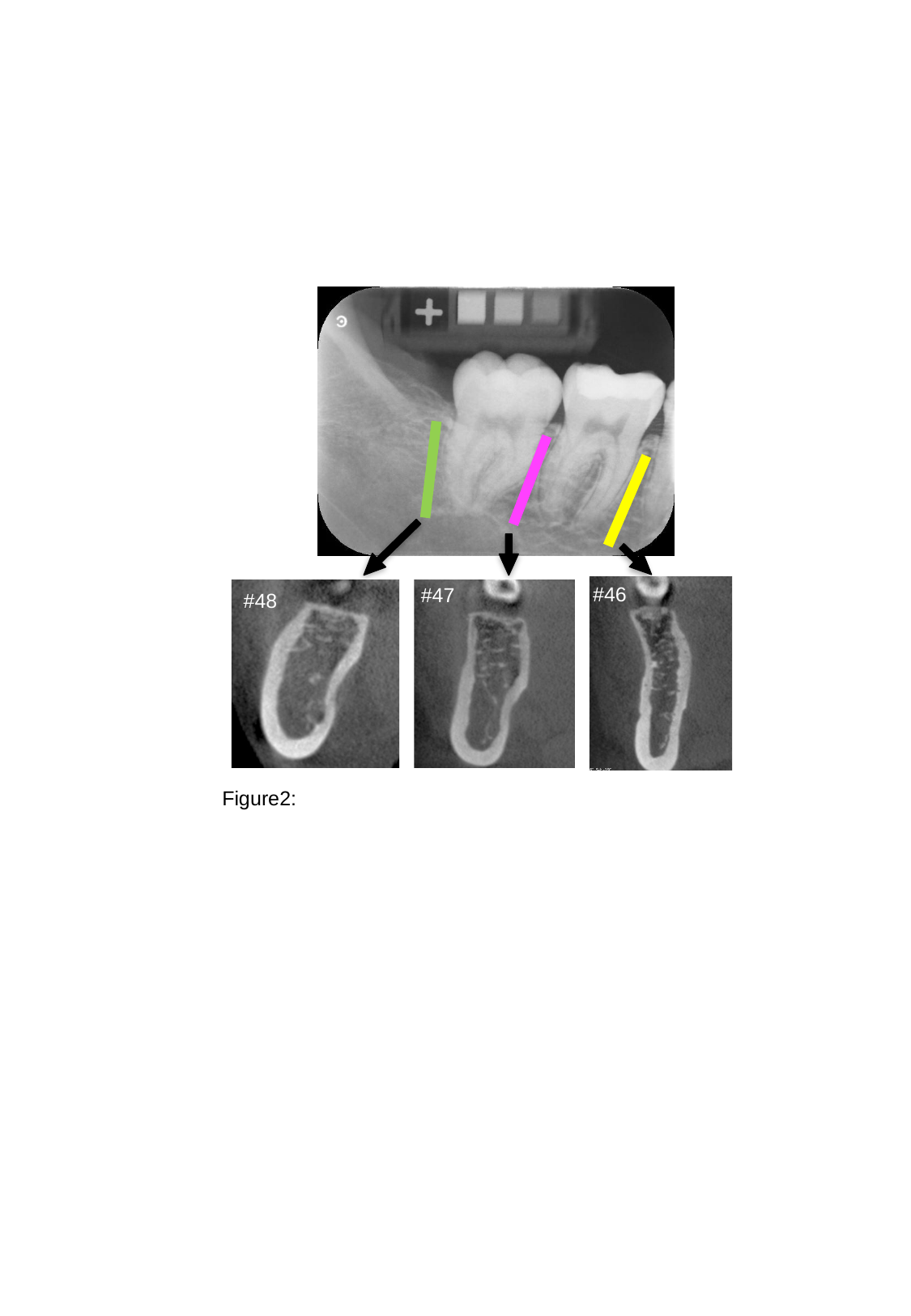

#46
#47
#48
Figure2:

## Slide 3
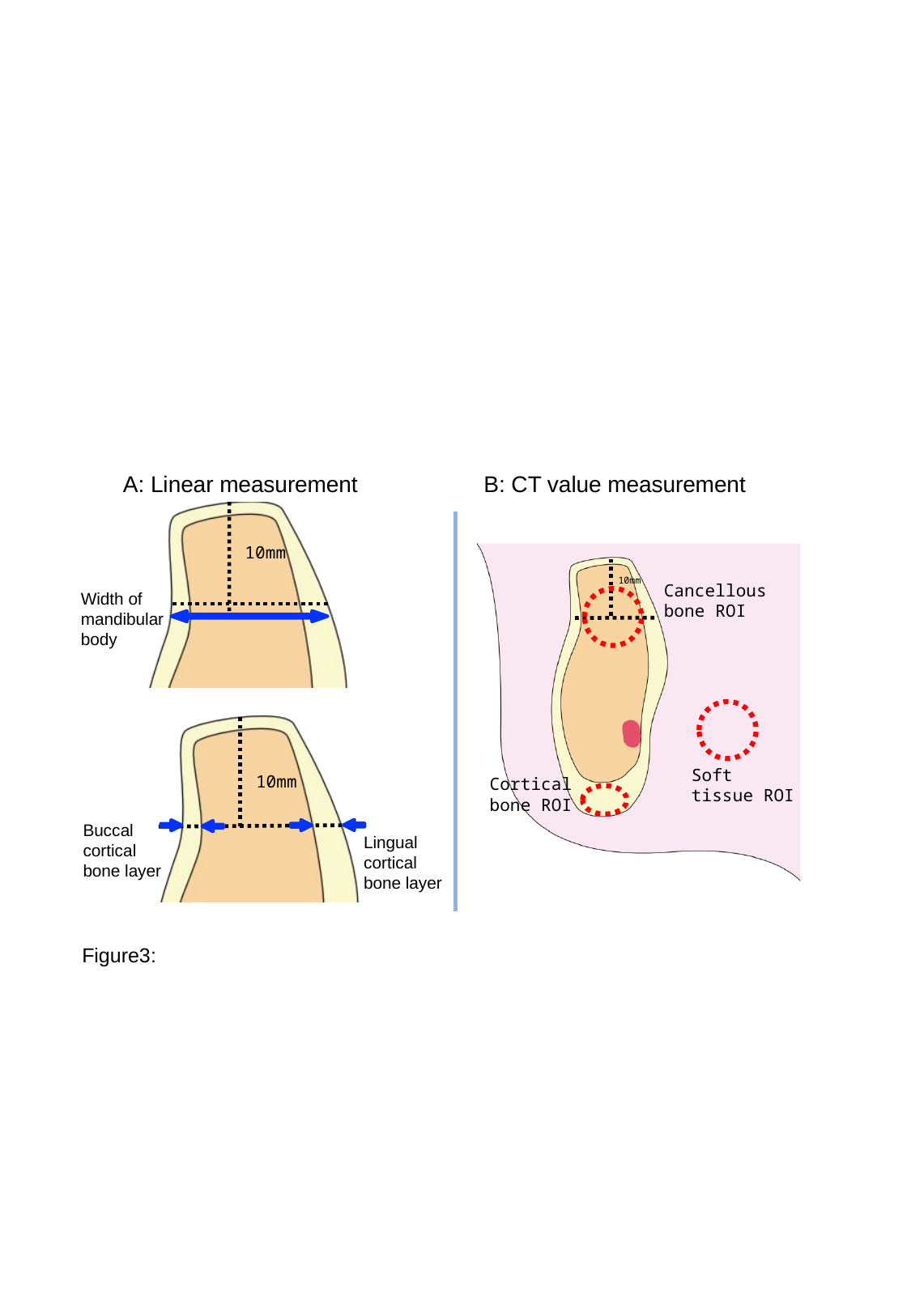

B: CT value measurement
A: Linear measurement
10mm
10mm
Cancellous bone ROI
Width of mandibular body
10mm
Soft tissue ROI
Cortical bone ROI
Buccal cortical bone layer
Lingual cortical bone layer
Figure3:

## Slide 4
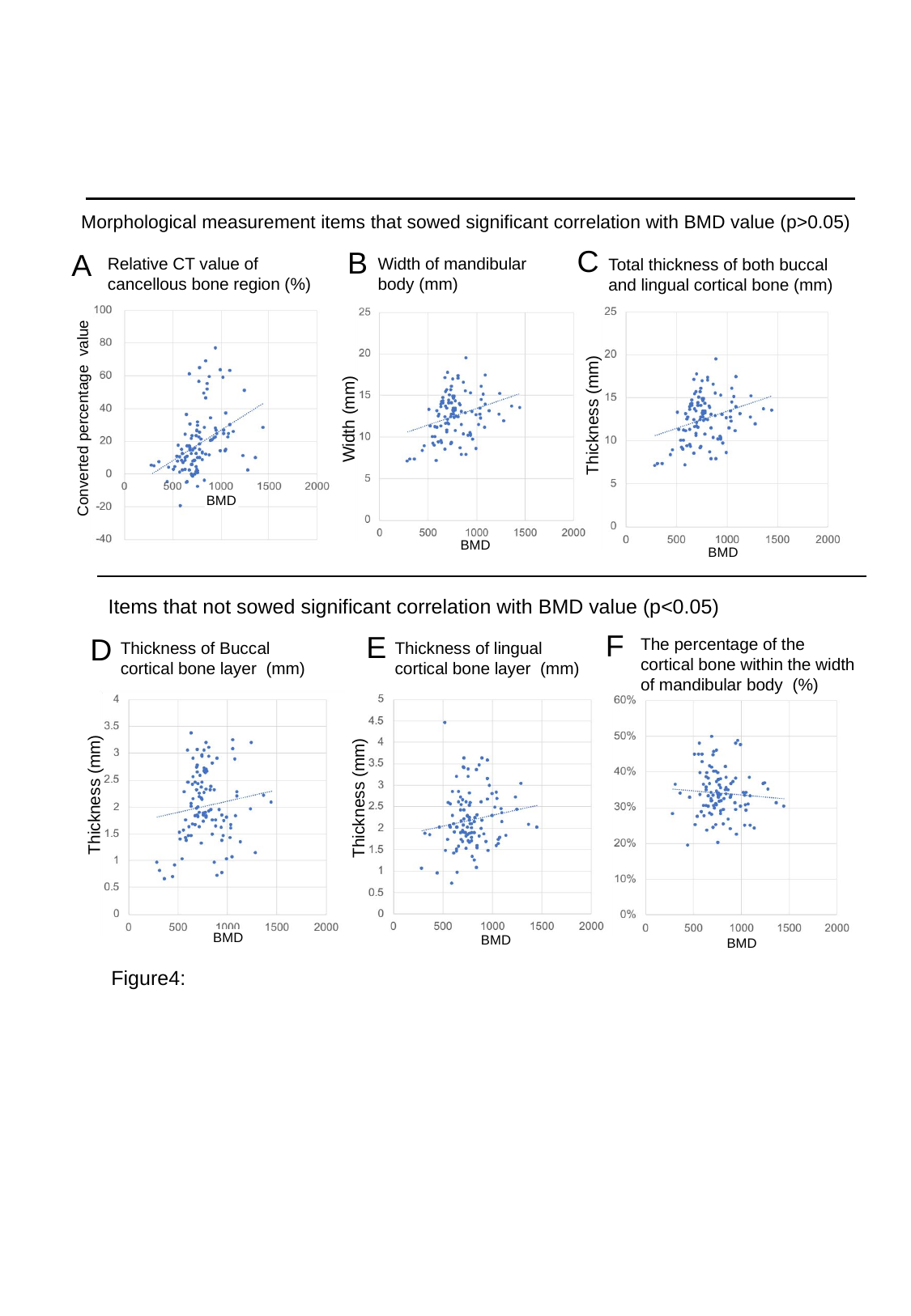

Morphological measurement items that sowed significant correlation with BMD value (p>0.05)
C
B
A
Relative CT value of cancellous bone region (%)
Width of mandibular body (mm)
Total thickness of both buccal and lingual cortical bone (mm)
Thickness (mm)
Width (mm)
Converted percentage value
BMD
BMD
BMD
Items that not sowed significant correlation with BMD value (p<0.05)
F
E
D
The percentage of the cortical bone within the width of mandibular body (%)
Thickness of lingual cortical bone layer (mm)
Thickness of Buccal cortical bone layer (mm)
Thickness (mm)
Thickness (mm)
BMD
BMD
BMD
Figure4:
